# Performance management in times of change: experiences of implementing a performance assessment system in a district in South Africa

**DOI:** 10.1186/s12939-018-0857-2

**Published:** 2018-09-14

**Authors:** Nonhlanhla Nxumalo, Jane Goudge, Lucy Gilson, John Eyles

**Affiliations:** 1 Centre for Health Policy, School of Public Health; Faculty of Health Sciences, University of the Witwatersrand, Johannesburg, South Africa; 2 School of Public Health and Family Medicine, University of Cape Town, Cape Town, South Africa; 3 Department of Global Health and Development, London School of Hygiene and Tropical Medicine, London, UK; 4 School of Geography and Earth Sciences, McMaster University, Hamilton, Canada

**Keywords:** Performance management system, Performance assessment, District, District health system, Human resources, Leadership, Management capacity

## Abstract

**Background:**

Health systems globally are under pressure to ensure value for money, and the people working within the system determine the extent and nature of health services provided. A performance assessment (PA); an important component of a performance management system (PMS) is deemed important at improving the performance of human resources for health. An effective PA motivates and improves staff engagement in their work. The aim of this paper is to describe the experiences of implementing a PA practice at a district in South Africa. It highlights factors that undermine the intention of the process and reflects on factors that can enable implementation to improve the staff performance for an effective and efficient district health service.

**Methods:**

Data was collected through in-depth interviews, observations and reflective engagements with managers at a district in one of the Provinces in South Africa. The study examined the managers’ experiences of implementing the PA at the district level.

**Results:**

Findings illuminate that a range of factors influence the implementation of the PA system. Most of it is attributed to context and organizational culture including management and leadership capacity. The dominance of autocratic approaches influence management and supervision of front-line managers. Management and leadership capacity is constrained by factors such as insufficient management skills due to lack of training. The established practice of recruiting from local communities facilitates patronage - compromising supervisor-subordinate relationships. In addition, organizational constraints and the constant policy changes and demands have compromised the implementation of the overall Performance Management and Development System (PMDS) – indirectly affecting the assessment component.

**Conclusion:**

To strengthen district health services, there should be improvement of processes that enhance the performance of the health system. Implementation of the PA system relies on the extent of management skills at the local level. There is a need to develop managers who have the ability to manage in a transforming and complex environment. This means developing both hard skills such as planning, co-ordination and monitoring and soft skills where one is able to focus on relationships and communication, therefore allowing collaborative and shared management as opposed to authoritarian approaches.

## Background

Although all components of the WHO building blocks of the health system are deemed crucial to strengthening the health system, the health workforce is central to all health systems and remains key to improving health and health outcomes [1]. Ensuring motivated and supported health workers with the relevant capacity significantly contributes to attaining national and global objectives [2]. Performance Management Systems (PMSs) relate to this endeavor. An important component of a PMS is a performance assessment (PA). Often used interchangeably these terms require clarification. PMS is a global mechanism through which organizations “set work goals, determine performance standards, assign and evaluate work, provide performance feedback (or appraise performance), determine training and development needs and distribute rewards” [3]. A PA on the other hand is a process in which an employee’s performance is evaluated. Measures are then developed in order to ensure improvement. The mechanism aims to both inform employees on the status of their performance and identify their weaknesses. It enables managers to identify those who qualify for a salary increment and promotion, identify training and development needs, place employees according to their ability and formally document reasons for any punitive measures. The PA therefore complements the overall function of the PMS. Although on its own it may not have a significant impact on health worker accountability it does contribute to improved work performance [4]. Concern about not meeting expected performance standards can reportedly affect motivation and performance and consequently result in an employee denying and avoiding responsibility [5]. An effective PA system therefore motivates and improves staff engagement in their work. It is an important component in the PM process that can result in a motivated and productive workforce and potentially improving health care services [5]. Linked to the governance and leadership building paradigm, it is a bureaucratic accountability mechanism that allows institutional oversight checks and balances within the public sector. As a tool to enhance answerability between the different levels of the health system, it has an element of enforcement in the form of sanctions or rewards [1, 6]. Health systems globally are under pressure to ensure value for money and high quality services. Performance management, specifically the PA component is critical to ensuring the provision of quality services and the improvement of quality health care practice [5, 7]. The increasing interest in PMS has generated questions as to why they have achieved limited success in improving the performance of health systems in low and middle income countries (LMICs) [8]. The aim of this paper is to describe the experiences of implementing a PA system at a district level in South Africa. Recognizing that the mechanisms are part of a comprehensive PA practice it highlights factors that undermine its intention and reflects on aspects that can enable implementation to improve staff performance for an effective and efficient district health system.

South Africa’s history is deeply rooted in discriminatory laws that were based on race and gender [9]. Prior to the new democratic government, the country’s political, economic and land restriction policies stratified society mostly according to these distinctions. This manifested in the structural organization of social life, interaction, access to basic resources and infrastructure such as health services [9]. The depth of racial segregation in all spheres of South African society translated into significant inequities in education and health status.

Racial fragmentation of the health system and deregulation of health services was fostered through the establishment of administrative authorities for each racial group. This fragmentation was consolidated by the creation of homelands, called Bantustans, notably featured by barren land and lack of resources and infrastructure [9]. This was the Apartheid government’s policy of territorial and political separation based on race [10]. Fourteen separate health departments for each racial group were created. This included one for each apartheid homeland for the different indigenous groups which by the end of apartheid all functioned independently in different areas of the country. Moreover, there was differential funding of the different health departments with health services in the Bantustans being the most underfunded [9, 11]. This level of segregation translated into significant health inequities, i.e. inter-provincial and rural-urban differences in access to basic services and other determinants of health currently [9, 12]. Moreover, it prioritized tertiary/ curative services rather than preventative ones [13]. Post-apartheid consolidation of the different departments resulted in a unitary department of health. Reform also resulted in three spheres of government that formed the basis for the division of functions within the health system. The National Department of Health has the responsibility of making national legislation, policy and establishing norms and standards of health services. Provincial departments focus on planning, regulation and providing comprehensive health services, except for environmental health services which remain the responsibility of municipalities. It also plays a supportive role to districts ensuring that systems are in place to maintain quality. Local government districts are the centre of health service delivery focusing on primary health care (PHC) services. However due to lack of clarity on administrative limits, functions at these two levels often overlap [12]. Regardless of these changes and after 23 years of a democratic state, the legacy of apartheid continues to constrain efforts to transform its public service institutions and to ensure equity in development. Moreover, it has been recognized that there is an explicit disjuncture between these three spheres of government such that the intended policies of governance from National level translate differently at the local level.

To provide further context to the paper it is important to reflect on the organizational structure of the South African health system as it bears on existing management practices and routines. Described as an aspect that depicts formal reporting relationships within a system and how activities are integrated and coordinated [14], one of the relevant features of organizational structure are structural elements such as centralization (of decision-making) and rule enforcement, as well as the more cultural elements such as management styles [14]. Although decentralization efforts to transfer management and decision-making responsibilities to the lower levels of the system are significant in health sector reforms, many processes remain largely centralized. This has largely been determined by history which also influenced the nature of management and leadership capacity in the current civil service. Recall that performance management systems are embedded in management and leadership paradigms. Although considered conceptually different, they often overlap and complement one another in practice. Management involves the more operational inputs such as planning, budgeting, problem solving and harnessing resources. Leadership establishes and communicates a vision and strategic direction for the organization to staff, including the softer aspects such as inspiring, motivating and linking individual goals to that of the organization [14–16]. In fact, the World Health Organization combines both these elements of inspiration and inputs and defines good management and leadership as: “providing direction to, and gaining commitment from partners and staff, facilitating change and achieving better health services through efficient, creative and responsible deployment of people and other health resources.” [17]. In light of the need to strengthen health system performance, managing and leading is critical [18].

During apartheid, managerial competence in the public sector was centralized and seniority was largely white and male. Moreover, public service practice was usually authoritarian, hierarchical, with rule-bound structures and procedures. This form of control has continued within current public sector bureaucracies and managerial structures [9, 19, 20]. These practices and structures need also to be understood as organizational culture. Although there are a myriad of definitions of the term, what is common across them pertains to the multiple aspects that are shared amongst people within an organization; such as values, beliefs, routines, sense making. Schien [21] sees it as ideas and practices “invented, discovered or developed by a given group as it learns to cope with its problems of external adaptation and internal integration”. New members are inculcated with this correct way to perceive, think and feel in relationship to their new work settings. Culture in this case is therefore used as a lens through which to understand and interpret an organization [22, 23]. It is within this organizational context that the most glaring challenge of the current health system is seen as the poor capacity to ensure efficient and effective human resource management (HRM), including weak management and leadership capacity. Concerted efforts to include black people and women into senior and top management positions have had negative repercussions such as the loss of institutional memory. It is part of the post-apartheid government’s endeavor to increase access for previously disadvantaged populations to the labour market. The translation of this corrective objective in provinces and districts has called for more attention to improve management capacity. The legacy of South Africa’ history manifests across all of its nine provinces of which one is the study site; the Gauteng Province. Further details on the province are provided below.

In post- apartheid South Africa since 1994, efforts to strengthen the South African health system emphasize the improvement of public sector management. The decentralization of legislative and administrative roles has rendered this call even more crucial at the lower levels. Although the establishment of the district health system and concomitant emphasis on PHC has increased access to health care, the poor performance and questionable quality of service delivery remains a challenge for the state [9]. Much of this has in recent years been attributed to weak management and leadership capacity; both inside the health sector and outside of it [24, 25]. Current health reform initiatives require strong leadership and capable management. It entails managers that are equipped with hard/operational skills. These are to provide the more technical support but also the soft/interpersonal skills which involve effective communication, coaching and hands-on support of weaker staff in order to address the multiple policy priorities and demands [10, 26, 27]. In order to strengthen capacity for improved health system performance there needs to be a focus on the PA component of South Africa’s existing Performance Management System (PMS) – called the Performance Management and Development System (PMDS). Consequently, this mechanism requires the aforementioned management skills to enhance the performance management process [28, 29]. A health system with a health workforce not nurtured by effective HRM processes such as the PA system is likely to fail to perform optimally. It is true that performance management in general is applicable and relevant across all sectors. It is however important to question whether the approaches in often profit-driven non-health sectors can be applied in health – a non-profit driven entity that presents with unique characteristics that may require different management approaches [30]. In a non-health sector there is rigidity and control where people are mostly required to perform prescribed and sometimes repetitive tasks, requiring structured management approaches. The health sector on the other hand mostly uses employees or contractors as providers in a system which makes judgements and citizens as users/recipients of the system presenting with different circumstances (or conditions). The nature of the work of providers therefore offers a great deal of variability. The extent to which services are delivered and the quality thereof rely on the extent of individual enthusiasm and motivation. In light of this aspect debates regarding PMSs specific to the health system need to consider the crucial and central role of ideas and practices of providers as well as patients [31]. This is more so because progressive performance management practices evidently contribute to improved patient outcomes [32].

As much as PA processes provide a foundation for training and development, motivation and enhanced accountability, it is crucial to explore the factors that enable and/or constrain effective implementation. Furthermore, although there have been several evaluations of the PA processes in the public sector, little research has focused on the health sector and few studies have examined PA in detail. It is against this context that this paper aims to examine and understand the implementation experiences of the PA component of the PMDS in a South African health district. It further tries to understand the factors that can improve its implementation.

## Methods and context

Our paper is based on data that was collected as part of a range of research activities aimed at understanding the micro-practices of governance in a South African district. This was particularly in the midst of health system reforms such as decentralization, revitalization of PHC and the establishment of a National Health Insurance (NHI). The research employed action research through a ‘learning site’ (further details can be found in [33, 34].

The study is based at a learning site in a district, District A, in a South African province, Gauteng. Although the smallest province - only covering 1.4% of the country’s total land area, it is known to be the economic hub of the country. It is home to more than 12 million inhabitants accounting for over 22% of the national population [35]. Despite its status as a better resourced and wealthier province it is faced with social problems that plague the rest of the country. The most pronounced issues are poverty and inequality in the distribution of resources and opportunities [36]. Due to apartheid urban development policies, there is an increasing housing shortage and an uneven distribution of basic services and facilities [36]. Leaving distortions in the spatial structure of the Province, the more affluent formerly ‘white’ cities are surrounded by poorly resourced townships and mushrooming informal settlements [37]. Gauteng is the second largest employer of civil servants with most of them in health, education and welfare [38]. Although considered a modern construct with a newly established public administration, its public service inherited functions, assets and personnel from former racially based administrations which had their own organizational cultures, procedures, and legislation and policy measures [36]. As in all the provinces, Gauteng has made efforts to integrate local and provincial health systems at the district level; however this has not been without challenges. The previous differentiation of salary and conditions of employment according to a particular employment body continues to constrain the employment of health care staff under a single health structure [9, 12, 39]. District A experiences the same challenges. Although the district was established in the year 2000 it still grapples with a staff establishment that is distributed between the employ of local and provincial government; rendering management and resource allocation a challenge.

District A was selected because the researchers and the district managers have a history of collaborative engagement which has generated a variety of collaborative research and capacity development initiatives. It constitutes three sub-districts all with diverse geographical and socio-economic characteristics – with a combination of rural, semi-urban and urban features. As part of decentralization, the district is undergoing development through increased infrastructure and transfer of administrative and management responsibilities as per delegation of these functions. The sub-districts are still in the process of development. The research was conducted mostly in one of its more developed urban sub-districts as that is where more of the pertinent study participants were based. The ‘learning site’ approach fosters in-depth learning and collaborative work within a specific geographic area. However because it allowed us to immerse ourselves in the local health system we also explored the other levels of the system that have an influence. The lessons learned can therefore be relevant across different settings in South Africa and in LMICs with similar contexts.

Data collection included in-depth interviews, participant observations and reflective engagements which took place between 2015 and 2016. Study participants were 26 managers at district and sub-district level which included senior managers, middle level managers and facility managers (Table 1). The senior managers at the district level were purposively selected as they were responsible for the different departments that constituted the district health system. This included those in health and non-health departments. The facility managers were selected through convenience sampling as available research resources limited geographic coverage. The study focused on the district and sub-district levels primarily because their managers were part of an action learning research of providing management support. These levels also enabled us to identify and understand how one level influenced management practices of the other. The Province was not included in the study design but senior managers provided insight of its influence. The majority of participants in the study were women. This is largely because nurses form a majority of public health workers and play a crucial role in the provision of health services particularly at the PHC level [40]. Managers are often promoted on the basis of clinical expertise in South Africa as in the rest of the world [41], so a large proportion is largely made up of nurses, predominantly a female occupation. Senior managers had managerial roles at the district level while middle level managers were responsible for management and supervision of facility managers at the sub-district level and reported to the senior managers. Facility managers had overall responsibility of running the clinics. The interviews explored the experiences of managing and being managed including experiences of implementing the PMDS. Reflective engagements were conducted with the senior managers with whom we have had prior interviews. We presented the findings from the previous interviews, allowing the managers and the researchers to discuss and reflect as to whether they were a plausible representation of their perspectives. This engagement enabled us to explore the dynamics and factors of management and performance management processes, feeding into the subsequent set of interviews. Reflective notes formed part of the data. A variety of monthly meetings were observed in order to get insight into the interactions amongst and between managers and staff. The observations also explored the management styles used by managers which created a link between their own reflection of their management and what played out in their interactions with their staff. Although not all the observations were conducted with the same managers that were interviewed, they provided an overall perspective of the institutional management styles that influenced the managers.

**Table 1:** District and sub-district managers interviewed

Participant	
Senior managers	11
Middle level managers	8
Facility managers	7
Total	26

**Fig. 1:**
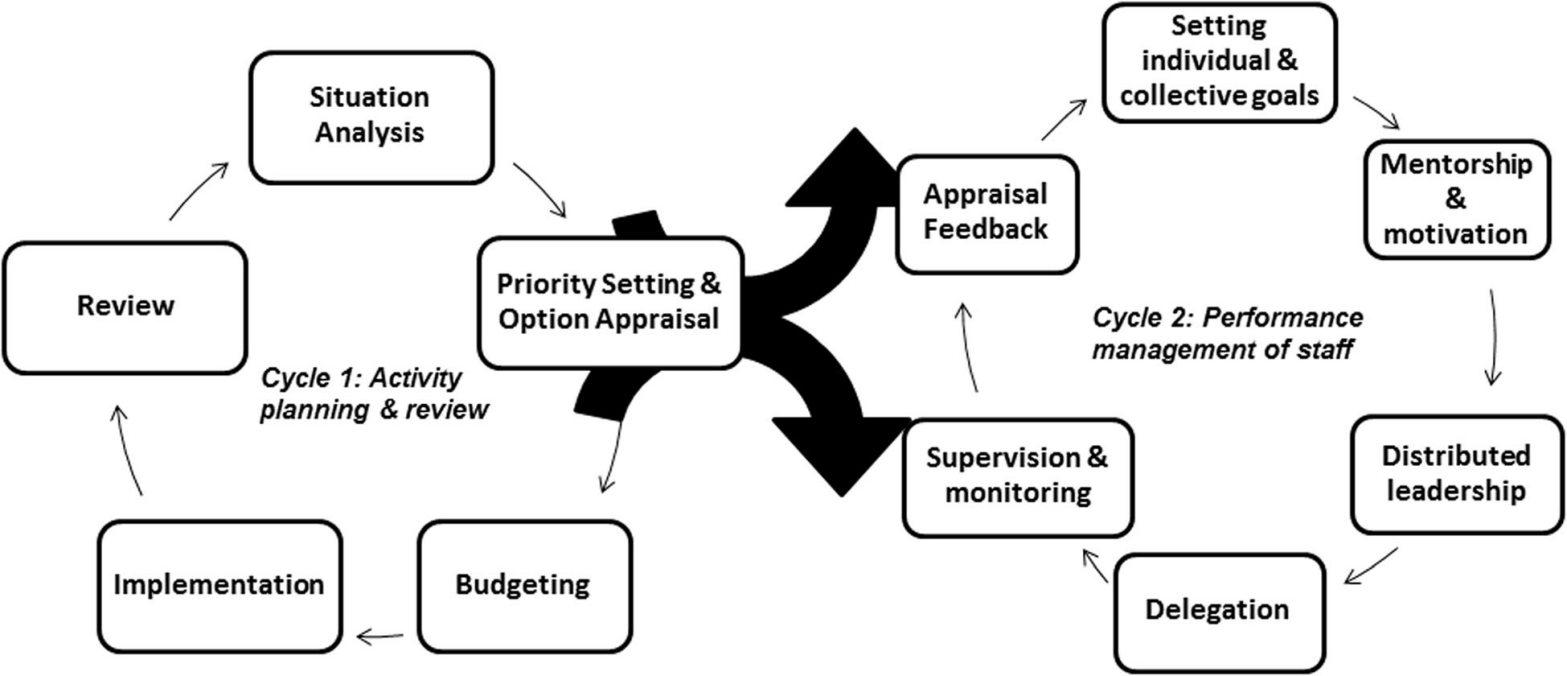
Conceptual framework adapted from Green [43]

The study used a case study design – an approach which allows an inquiry of a phenomenon within its own context [42]. Performance management processes are influenced by the context within which they function and a case study approach enables us to construct an understanding of contextual influences. This study was also guided by a framework adapted from Green [43] (Fig. 1) which suggests that the routine practices of governance (such as performance management) constitute two inter-dependent cycles. The first (Cycle 1) is the activity planning and review i.e. planning, budgeting, implementation and review of expenditure and achievement of process and health outcomes, while the second (Cycle 2) is the performance management of staff which includes setting individual & collective goals, mentorship & motivation, distributed leadership, delegation, supervision & monitoring, appraisal and feedback. This cycle (Cycle 2) requires strategies to enable the creation of spaces for discussion where alternative views can be expressed and acted upon; reduce the length of the ‘delegation chain’ in which rationale importance and motivation is lost; maintain motivation and develop short feedback loops to allow staff to understand the influence of the performance and alignment between organizational and individual goals. It is the alignment between these two cycles that facilitates plans to be translated into effective implementation. Although this paper focuses on the second cycle, it is part of a larger action-learning study that aims to understand how they function in the study district so as to support and improve governance practices and accountability in the health system of South Africa. The overall study aimed to understand the daily practices of governance in the study district. It therefore explored the health managers’ experiences of other aspects of governance such as financial management at district level. Through a thematic content analysis that was conducted through an in-depth iterative process with a team of researchers, transcripts, reflective notes and field-notes were analyzed to identify a priori and emergent themes. Identification of the routine practices and challenges of PMS in the district followed an inductive and deductive process. Data was examined against the original objectives of the study to identify predetermined themes while any divergent themes were examined by returning to the data. The iterative process of reflection and triangulation of the range of data ensured the trustworthiness of the final analysis. Data from the interviews and the reflective engagements are represented through quotes while field notes from the participant observations were used to inform and confirm the themes identified.

Ethical approval was obtained from the Gauteng Department of Health and the study district Department of Health. Approval was also obtained from the Committee for Research on Human Subjects at the University of the Witwatersrand. Informed consent was obtained prior to any data collection and participants were given the opportunity to decline to be interviewed without prejudice. Pseudonyms have been used for the names of the geographical places described in the paper.

## Results

This section of the paper firstly describes the participants in the study. We provide a description of the PMDS in South Africa and the prescribed implementation. We then describe how it is implemented in reality and provide factors that influence implementation.

### Description of study participants

The majority of participants were female with only 7 males. Almost all the female participants had been qualified as professional nurses with PHC training except for three participants whose roles were in support services such as Human Resources, Finance, Procurement, IT etc. hence with commerce-oriented qualifications. Most of the male participants’ qualifications were related to their positions in support services. Senior managers had the longest experience ranging from 20 to 25 years. Three participants were close to retirement by the time of data collection with over 35 years’ experience. Most of them had been in their current positions for over 5 years while one had just been appointed with 6 months in the position. The majority of senior managers were promoted on the basis of their clinical experience; hence had limited training in management while those in support services indicated that they received training during their career. The middle level managers all had more than 15 years’ experience while most of them had been in their current positions ranging from 2 to 3 years. Only one was in their position for 6 years. All of the facility managers had over 20 years of experience in the profession; however most of them had been in their current positions for 1–2 years. Only one facility manager had been in their current position for over 10 years. Almost all the middle level and facility managers indicated that they had no training in management. Only one middle level manager reported that she an opportunity to attend a formal training course before being appointed to her current post out of her own volition.

### The performance management and development system in South Africa

As part of a series of mechanisms to improve public service performance in South Africa, the PMDS is meant to create a link between the performance outputs agreed to with the individual to assess and their performance [44]. Conceived as a compulsory national accountability framework in the public sector, the PMDS is a tool to enable managers to ensure planning, the monitoring of progress and assess outcomes. It ideally involves a continuous iterative face-to-face interaction between the supervisor and employee. An annual cycle allows for the mutual tracing of the employee’s performance [45]. The 12 month period cycle culminates in a performance assessment where the supervisor and manager agree on a score; ranging from 1 (unacceptable performance) to 5 (outstanding performance). The higher score qualifying supervisees for an award [45].

### Implementation of the performance management and development system

#### Informal implementation

Although some respondents had a positive view of the PMDS in that it facilitated learning and development: *“I think for me… because I have been a manager in a clinic and I was managing 100 people…I think it develops people a lot”* (Middle level manager^1^ 14), the majority expressed the effects of weak management on the PMDS process. For instance, it was noted that the process included informal practices such as the granting of scores that ensured an award without merit:

*“So sometimes you’ll find that a person [who has been delegated to appraise other staff] has rated a person high. And then normally I don’t accept it. I normally say okay, can you tell me what this person did to get a five…Then the person will say ‘no, no, he’s a hard worker’. I’ll say, when we talk about five, you must tell me that the person has gone an extra mile. But if a person is doing the norm, you cannot give them a five.”* (Facility manager 6)

Furthermore, due to the lackluster approach most felt that the process was a meaningless exercise where one did not expect any opportunities to develop skills or career development:

*“The PA [Performance Appraisal] system here is not a big deal. It is not serious at all. Mrs X just sits with me and says ‘Ok. I’m giving you a 3 here here and here.’ That’s it. No room to say, you are not doing well here, what could be the problem. There is no chance to develop yourself or where they (managers) say, you need development on this …or you need to attend this course etc.”* (Middle level manager 13)

As the practice of PMDS has limited integrity many were of the view that it has affected other components of the process such as training in that it is equally not applied meaningfully*: “There are workshops that we see and we go to…but not those that are paid by the department or even related to the PA development process. It is usually just a random workshop that has become available and we just go.”* (Middle level manager 13).

This implies that the PMDS process was autocratic in nature and therefore, potentially, lacked meaningful discussion. Consequently it became routinized such that it was often implemented as a matter of compliance.

Several respondents indicated how managers failed to raise issues regarding performance (particularly when poor) throughout the year, such that it became difficult to raise them in the performance assessment process. Consequently, this undermined the process: *“The manager would be too scared to sit with the person and say ‘but I don’t think you are a 3'…because during the year they have not discussed your issues with you. Now at the end of the year they cannot say ‘what you’re doing is not good’ ”.* (Senior manager^2^ 11).

This was possibly an indication of how the performance process is considered a separate function from overall management. Ideally, the process should occur throughout the year.

In addition, a few senior managers expressed a concern that a precedent was established in the district where there was reportedly an initial pattern of awarding high scores to everybody. This practice seems to have remained and managers consequently found it difficult to assign different scores that were a true reflection of the staff members’ performance. One manager said:

*“This whole group of people will on a continuous basis get fours and once you’ve given a four it’s very difficult to go back and say but now you should get a score of XYZ… because you’ve been doing things the same way”.* (Senior manager 11)

This established practice undermined the assessment process such that the system could not accommodate changes or revisions.

As a symptom of poor management skills, several respondents indicated that managers found it difficult to manage relationships and often preferred to protect them to avoid conflict. *“Perhaps the two of you might have not have been objective… because of the very close relationship that you may have. It results in ‘I don’t want to trample on his/her toes’…Then you say for the sake of peace, let me give a four …for example…To avoid fighting just give people a four, it is a system that doesn’t work.”* (Senior manager 1).

This is an indication of how the PMDS does not often allow for relationships to be nurtured through meaningful conversations linked to performance and mentorship. Instead, it is used to maintain existing relationships. This interestingly contrasts with the autocratic approach of conducting the process noted by other managers.

Most of the managers noted that poor conduct or performance was not adequately dealt with if at all, hence staff conducted themselves with impunity: *“You cannot hold anyone accountable. Things don’t get done and there are no consequences here. People don’t get punished or fired. People do whatever they want. If they do wrong…they get away with it…then you come in (as a manager) and want to achieve your targets…but the people that are supposed to do the work do not do it… That is why [District A] is struggling to meet the TB care rate target.”*(Middle level manager 13).

One can therefore surmise that the PMDS does not necessarily enable managers to act on poor performance in that people are not dismissed despite lack of delivery. When asked about the consequences of poor performance after a PMDS outcome one manager indicated that: *“There is demotion yes. But…not dismissal.”* (Senior manager 1).

The PMDS evidently does not provide support to the overall management system such that managers have no confidence in its ability to enable them to act on performance issues such as enforcing discipline or dismissal.

#### Financial rewards and lack of accountability to budgets

Several respondents noted that there was an established culture in the PMDS process which was particularly based on financial incentives such that it undermined the developmental aspect of the process:

*“People are more focused on the bonus…on the money and not on the development…. People are more accustomed to a bonus. Now they were asking me when are we getting paid for PMDS?”.* (Facility manager 7)

Furthermore, a few respondents pointed out how managers allowed subordinates to rate themselves and accepted the self-rated score without any inquiry or engagement thus showing lack of accountability to other components of the mechanism such as the budget:

*“It is standard practice with PMDS for the person being supervised to rate himself. I think I’m a four or I think I’m a five and then it will go to the manager and the manager … because in any case they all get (an award)… doesn’t care and would probably sign off on that”* (Senior manager 11)

The PMDS process has been compromised due to a range of practices and this has undermined its original intention. The improvement of management skills has the potential to enhance the process so that it begins to achieve the goals intended.

### Factors influencing implementation

#### Organizational culture

It was interesting to learn how a historical feature of management has influenced current approaches in the public sector. One manager reflected on a ‘command and control’ approach to managing others and how this was influenced by the experience of discipline in all spheres of government during apartheid:

*“I grew up in a school where it was very disciplined. I went to the army and you know how those army years were, it was terrible. It was very bad. It was very disciplined. So you grew up in a disciplined environment and it’s something that becomes a part of your life.”* (Senior manager 9)

It was evident that this approach of instilling discipline influenced the PMDS process. Many respondents were of the view that it was punitive and was merely introduced to monitor people. Some managers noted:

*“The PMDS is a way of the managers to punish the workers”. (*Facility manager 16)

*“I deal with it [underperformance] immediately. It’s the targets, they must explain why. What is their reason for not reaching that target and what is their improvement plan that they are going to do. “* (Middle level manager 17)

These views suggest that historical authoritative and autocratic practices still influence current management approaches as much as they manifest in the performance assessment practices. This legacy is also reflected in the top-down decision making processes of the health system where much of the decisions are made at national and provincial level.

A respondent noted how this has resulted in those below responding to demands with limited collaboration and/or mutual exchange:

*“The head office [Province] thinks that we exist for them, so when they want information they use us as information taps” Instead of US saying ‘these are the issues’ and then THEY should come down to support. So it starts from there… The manner in which we do things – it’s a typical bureaucratic thing”* (Senior manager^ii^)

Several respondents noted that top-down decision-making processes resulted in policies that did not speak to the reality at local level. One manager reported on the difficulty of implementing policies designed through a hierarchical process and how they are often far removed from the context in which they should be implemented:

“*There are quite number of policies or circulars which the province is issuing, which at times I find myself asking… hmm why am I having difficulty integrating this? Why am I having difficulty in implementing this? It appears simple to implement from the paper but not practically …Remember the head office [provincial office] has just but a handful of people. They do not deal with these things in practical terms. WE are dealing with them on practical terms, and we need to implement practically.”* (Senior manager 1). Moreover, they indicated that the hierarchical structure and the distance it creates between levels of the system reportedly hinders and/or slows down implementation processes: “*It is the hierarchy. We don’t have a flat structure. It’s hierarchical and problematic. That is the only thing which troubles me. We delay to implement things because of the decisions that are made above.”* (Senior manager 1).

This has manifested itself in the engagement between supervisors and supervisees such that it is common for one to be told what to do and not to challenge or question authority. One manager indicated this by noting:

*“No, you’re not allowed to ask. It’s your job. If they ask you to do it, then you do it. We’re too afraid to question it.”* (Senior manager 9)

Although senior management may issue autocratic instructions in order to get things done, without being able to adapt policy to the local context, managers closer to the front line may well resist such policies. The views above indicate how the top-down management approach across the levels of the health system manifests in other management practices such as the PA.

In light of the hierarchical nature of the system, the implementation of the PMDS did not vary across the different levels. All managers followed similar processes as prescribed; hence the experiences and perceptions of the process did not appear to differ between male and female participants. Yet there were differences between the facility managers and the rest of the managers across the district. It was noted by some of the facility managers that the outcome of their assessment was not a reflection of their own individual performance. Some noted that the performance of the facility reflected back to their line managers (the middle level managers) and therefore they received a similar score as they did. One facility managers explained as follows:

*“You know the issue of PMDS on my side, it’s a problem… because she (middle level manager/line manager) said, if I cannot get the PMDS (a bonus) you’re not going to get it also. If my manager is not going to get the PMDS, I’m not going to get it either….because for her (middle level manager) to get the score, it’s through the performance of the facility. If her facilities are performing, then they will say your facilities are performing, they are reaching the targets, then she’ll be able to get (the bonus). So immediately when we are not performing, there is no way that we are going to get the PMDS (bonus).”* (Facility Manager S6)

#### Patronage

The district reportedly made the decision to recruit from within the area as part of the broader provincial imperative (embedded within State policies to address economic and skills inequities) to create opportunities for employment and build skills in the community. This practice is the manifestation of the social and cultural values of the broader South African society where there is the inherent belief that there should be collective benefits of a new democracy. Several respondents however indicated that this compromised management processes because it created spaces for patronage where managers were familiar with recruited staff. A manager was of the view that it encouraged inequity in the meting out of awards, often resulting in the awarding of those who are undeserving, which affects the morale of the staff overall. *“That’s patronage…you can’t allow patronage to everybody. There are those whom you don’t give; there are those whom you give… so it has an effect on the morale of the staff. There are those who get bonuses, whilst they are not working. There are those who don’t get bonuses, whilst they are working.”* (Senior manager^ii^).

This implied that the relationships between supervisors and supervisees are likely to be compromised by a culture of patronage. A manager is faced with a situation where s/he has to supervise staff from the same community. This compromises their ability to manage in fear of damaging the relationship or creating conflict. This affects the extent to which they can maintain professional relationships.

#### Training

The majority of respondents indicated that lack of training and/or induction for newly appointed managers with no prior skills of management contributes to the limited capacity of management and leadership. Consequently this results in the poor ability to manage others and provide mentorship. One manager said:

*“So a person was just a mere clerk and is now a senior clerk. They need to be empowered and skilled in managerial skills and supervisory skills and attitude. So you’ll find that they just get promoted. They’re not getting training. They’re not orientated and all that…and they start to mismanage their employees…which is a problem.”*(Senior manager 5)

Another manager added that:

*“You find that you put a person in a position and you put him under pressure, and you actually set him up for failure because he has not gone to any training or hasn’t been developed with regard to managing people.”*(Senior manager 11)

A few respondents indicated how limited skills due to lack of training also resulted in managers that cannot facilitate difficult conversations and situations. One manager noted:

*“What I see are managers or supervisors who don’t want to take responsibility and accountability in terms of their own subordinates. Because they will come to you with a simple question ‘The person [their subordinate] is not coming to work. What should I do?’ … But as a manager they must be skilled on that. And that on its own it tells me that this person lacks the training on employee management.”* (Middle level manager 2).

This indicates that the district appoints managers with limited management skills without providing tools and/or mechanisms to support and develop their skills. This is likely to compromise the management of people and the performance of the district. It can potentially result in managers who do not assume the responsibility to manage and be accountable for the district’s performance.

Managers related their experiences of relationships with their own managers, indicating that they had to deal with what they alluded to be difficult personalities.

A manager expressed this as follows:

*“My immediate supervisor…[takes a pause…as if hesitant]…she is a nice person…but she is not a manager. I don’t know… It’s a personality thing. She has tantrums…she is not consistent. Today she is this way…moody. Tomorrow she is that way. For instance, we will all have a meeting and we will agree on certain things…then when it is escalated to a higher meeting…and we are sitting there, she will say something completely different…and accuse one of us…and say she never said this… It is not only me. We all feel that way…and it is one of those things that are spoken about but it stays the same”* (Middle level manager 13)

These narratives indicate that a series of factors such as lack of support and training and a culture of patronage result in limited abilities to manage people in an organization. Furthermore it indicates that when functioning in a hierarchical system where the middle managers’ own managers employ a command and rule approach with limited consistency they mirror a similar managerial style. All of these factors translate to poor performance management practices.

#### The role of communication and relational management approaches

One manager however had an interesting approach to management that had the potential to improve the implementation of the PMDS. Their view indicated that using more communicative and relational management skills in everyday routines can inform and improve the PMDS process. The manager explained how she approached managing difficult relationships amongst staff:

*“With regard to managing the people, when I came in there was a lot of negativity towards (the department). There was a lot of bad attitude from people within (the department). I have now given them training on the code of conduct. We’ve had quite a number of meetings where we had to put out fires between different people. You’d find that they like screaming at one another but that hasn’t happened for quite a while though. This specific lady said to me she’s changed her attitude. She now doesn’t say I cannot do something, she would say but what would ‘senior manager 11’ have done. Because I try to teach them that even if you can’t, try and see if there’s not a plan B. But then slowly you can start to see that there is a change also in the relationships. I think yes we are starting to be a team instead of us always fighting one another.” (Senior manager 11).*

The manager highlighted the value of incorporating management practices in everyday routines in order to reestablish relationships and ensure continuous learning amongst staff. It is a better process than addressing performance issues during performance assessments that occur at specified times of the year. Instilling this form of management has the potential to improve how managers and those that are managed approach and view the PMDS process.

#### Unions in the public sector

The strong presence of unions in the district has shaped the way in which public servants relate to management. Unions evidently exert a strong influence in the management practices in the district. Most of the respondents were of the view that people had the tendency to refer management issues to unions hence managers were reluctant to manage staff as required:

*“Managers cannot be assertive, because they are concerned about the unions”.* (Senior manager^i^)

*“The public sector is highly unionized. What has been happening in institutions is that the unions appear to be overpowering management. In fact in terms of management, unions are managing. They are co-managing”. (Senior manager* 1)

*They (unions) are giving facility managers problems to manage. Let me make an example…concerning your performance. You feel that it’s not right. You say ‘She’s (my supervisor) judging me. She’s not objective.’ Then you will go to the union. Instead of the union hearing the other (supervisor) side, they will come and take your side. It’s killing people from performing. People are not performing because the minute I confront you (about your poor performance), you go to the union.* (Facility manager 19)

Not only do managers lack the capacity to manage people, support mechanisms within the district and factors such as unions compromised the extent to which they can. This evidently affects management processes with adverse implications for implementing performance management and subsequent measures to address any deficiencies identified by the supervisor and supervisee during the PA process.

## Discussion

A summary of findings in this paper highlights key organizational and contextual factors that affect the implementation of the PA of the PMDS in the district health system. The dominance of hierarchy where there is an autocratic approach continues to influence the management and supervision approaches of front-line managers. Management and leadership capacity is constrained by a myriad of factors such as insufficient management skills due to lack of training. The established practice of recruiting from local communities facilitates patronage - compromising supervisor-subordinate relationships. These findings are further discussed below in relation to the broader national and broader literature.

### The tension between social and organizational cultures

Martinez and Martineau [28] assert that performance management systems in most instances do not take into account contextual factors in developing countries, therefore undermining implementation. Similar to our findings regarding the influence of historical organizational culture, other studies have reflected that cultural and traditional dynamics, that is, the ‘old ways of doing things’ can permeate the workplace such that they have an effect on performance management practices such as PAs. Culturally-related work values can therefore affect one’s interpretation of a performance dimension [8]. Culture closely plays a role in that social practices conflict with administrative rationality; an aspect that is central to organizational management [8]. The managers in our study cite instances where a supervisor is faced with disciplining a peer from the community, hence conversations about performance are compromised and difficult. In the Ghanaian context, people found it difficult to be critical of others’ performance while in a face-to-face situation noting how they were unable to advice subordinates of their poor performance [8]. Further demonstrating how the external factors such as cultural norms influenced management and processes in the workplace, Ghana’s traditional ethos renders a society which places collective values over western-influences of individualism [8, 46]. A similar dynamic was at play in our findings where the district’s recruitment processes were fostered by drawing from the community as a means of sharing the benefits of a new democracy, hence empowerment through employment. South Africa’s employment equity policies are geared toward meting out the ‘collective’ benefits. This needs to be taken into cognizance when considering performance management practices.

### Management and leadership in the South African context

Defined as “patterns of shared values and beliefs over time which produce behavioral norms that are adopted in solving problems” [47] organizational culture has a significant impact on management overall as reflected in our findings. Taking root from apartheid practices of authoritarian, autocratic and paternalistic management – and manifested through hierarchical state structures - the current South African health sector has inherited and even internalized this management approach. In their study of public hospital management, Von Holdt and Maserumule [48] asserted that this is more so in the nursing profession, which in those times was dominated by white nurses and bureaucrats, placed acute emphasis on discipline and status-driven values [48]. Organizational context manifests in the culture of reverence to hierarchy, hence final decisions are assumed to be the obligation of those higher up in the hierarchy. This ultimately removes the sense of autonomy and /or accountability for decisions or actions taken across all levels of the system. The managers in our study reflected on this context where the province reportedly imposes mandates through autocratic means as one manager’s accounted *“the head office, thinks that we exist for them*…”. Gilson, Elloker et al. [27] mention how this has rendered managers at the district too passive and fearful to assume decision-making authority. Seeing themselves as agents of those controlling from the outside – an “external locus of control” [27, 49], this results in district managers who focus on routine and procedure rather than relating with people.

One of Hofstede’s [50] dimensions of culture, generating particular behavioural patterns that influence people in any given context, is relevant. This dimension, uncertainty avoidance, leads to people avoiding taking risks and accepting change, also avoiding taking personal initiatives that are outside or divergent of the given roles [8]. Our findings describe a similar notion where the PA process became routinized and merely a ritual which is not considered significant or important. Moreover, finding it difficult to assert their authority and rather than allowing the assessment to determine the extent of performance through the administering of scores, they often awarded high scores to avoid conflict. This was also noted in Ghana where managers were reportedly reluctant to exercise assessments objectively and allocate genuine scores according to performance [8]. This notion of a domineering autocratic culture may appear contradictory to the cited reluctance of managers in our study to give out scores that subordinates deserve. However, this need to avoid conflict occurs within the practice of compliance which is an indication of obedience to authority. Although subverting the assessment process itself, they implement the practice due to compliance rather than due to the recognition of its intention.

#### Management capacity, the role of trade unions and training

Our findings indicate various factors that affected management capacity in the district. Efforts to address the injustices of the past in South Africa aim to ensure diversity in public service institutions. The enforcement of employment equity and affirmative action policies has created job opportunities for the previously disenfranchised Black population. It has however resulted in the attrition of a skilled White workforce. Left with limited skills the health sector is experiencing high vacancy rates and in certain instances the filling of those posts with personnel with limited prerequisite skills and experience [19].

The transition to post-apartheid South Africa was also coupled with an enhanced role of unions in public sector labour issues. During apartheid, Black public service workers were not permitted to join unions – a strategy for the apartheid state to exert repressive and abusive labour practices. The establishment of a new democratic government allowed for increased recognition of unions, thus formalizing their role in protecting workers’ rights. However, this has come with increased control and involvement in labour and management processes – limiting the ability of managers to manage and assert control and disciplinary measures. In a study at a Gauteng public hospital in South Africa, managers voiced their frustration with the dominant role of unions in running the hospital, rendering it difficult for them to assert authority and discipline [48], echoing similar sentiments from the managers in our study.

Several managers at district and at facility levels indicated that they occupied management positions without training. Findings from a survey on management in the South African health sector indicated the limited capacity of managers to lead in the health sector, hence the need for increased training in management skills [51]. The study confirmed a notion that has been alluded to in several studies in the South African public health system - that managers display limited confidence in their competence to manage [51]. Managers in another study rated themselves in a survey as reasonably competent but did not consider themselves to be at a sufficient level [24]*.*

### Implication for the practice of performance management systems

Our work highlights the importance of understanding how the interrelations between different elements inside and outside of an organization shape emergent patterns of behaviours and responses [52]. Understanding the impact of context and organizational culture should therefore be recognized when seeking strategies to strengthen performance management systems and more importantly management and leadership capacity. Findings in this paper indicate the need to shift away from management strategies that undermine the role of history, context and culture in influencing how a system responds to constant change and complexity. There is a need for the reevaluation and changes in organizational culture – as the notion of “reculturing” of an organization implies [27]. The recognition of the interconnectedness that exists within a system will foster the understanding that health system performance relies on the role of all actors across the system, hence a need to shift away from rigid and paternalistic notions of leadership. In this regard there is a growing sentiment for an increased role of communication and relationships within the public service in general. To further this argument there is the notion that middle level managers in their positionality of being in the coalface must understand that context plays a role in sense-making. This refers to translating constant change, conveying information and facilitating collective communication with other staff to generate ideas [27]. Efforts to improve performance management systems and inevitably performance assessments need to take into account these ideas when looking at supervision and leadership skills. Capacity development and training in this regard should instill elements of doing things differently. That is, where there is a recognition that modes of training and /or capacity development need to equip managers with the capacity to deal with complex systems that are shaped by the interconnectedness between context, people and relationships. Performance management systems that indirectly guide PA practices require processes that allow for mentorship, flexibility and recognition of complexity. Nurturing leadership and management skills that incorporate this understanding could potentially develop mechanisms that enhance performance – towards a responsive and resilient district health system in South Africa.

## Conclusion

Several factors contribute to the limited gains of PA systems. Firstly, organizations commonly underestimate the influence of contextual factors on the implementation, such as organizational culture, lack of political will, and limited reward and sanctions mechanisms [4, 8]. Secondly, performance assessments are often didactic, bureaucratic practices of allocating scores and assigning incentives according to the scores. They can become routinized and often lack the joint reflection on an individual’s performance that can identify where training and support is needed. Mentorship, training, and consequences to poor performance are often overlooked or not implemented which further undermines the process. Although a performance assessment is an important component it should be recognized that it requires a comprehensive range of performance management practices to effectively achieve the intended goals [28, 29, 53].

Our study shows that a range of factors influence the implementation PA of the PMDS in a South African district. It is evident that a large part is attributed to context and organizational culture but also to management and leadership capacity. In the aim of strengthening district health services in LMICs there should be improvement of processes that enhance the performance of the health system. Implementation of PA processes however will rely on the extent of management skills at the local level. In countries that seek to improve health system performance, there is a crucial need to develop a cohort of managers who have the ability to manage in transforming and complex environments. This means developing both technical and operational/hard skills such as planning, co-ordination and monitoring. It also requires developing interpersonal and communicative skills allowing collaborative and shared management rather than authoritarian approaches [24, 27, 28]. Improving management skills and capacity has the potential to influence organizational culture and management approaches that lead to an efficient, effective and resilient district health system.

### Limitations of the study

Although the study is based on the experiences of managers at the district level, it could have gained from the perspectives of managers at the provincial level. This would have potentially provided a better understanding of the extent of influence on management practices at local level. Due to the constraints of a limited research team it was not possible to explore experiences of facility managers in a larger sample of facilities. This would have provided rich data and broader perspectives to inform the conclusion of the paper.

## Abbreviations

NHI: National health insurance
PA: Performance assessment
PHC: Primary health care
PMDS: Performance management and development system
PMS: Performance management system

## Footnotes

1 Manager at the sub-district level

2 Manager at the district level

## References

[1] World Health Organisation. Monitoring the building blocks of health systems: a handbook of indicators and their measurement strategies. Geneva: World Health Organisation; 2010. http://www.who.int/healthinfo/systems/WHO_MBHSS_2010_full_web.pdf. Accessed 18 June 2017.

[2] World Health Organisation. Working together for health. In: The world health report, vol. 2006. Geneva: World Health Report; 2006. http://www.who.int/whr/2006/whr06_en.pdf. Accessed 20 June 2017.

[3] Briscoe DR, Claus M, Varma A, Budhwar PS, DeNisi A (2008). Employee performance management: policies and practices in multinational enterprises. Performance management systems: a global perspective.

[4] Lutwama GW, Roos JH, Dolamo BL (2013). Assessing the implementation of performance management of health care workers in Uganda. BMC Health Serv Res.

[5] Musyoka FN (2015). Performance appraisal influence on health workers’ performance in public hospitals: case of Mbagathi hospital, Kenya. The Journal of Global Health Care Systems.

[6] Cleary SM, Molyneux S, Gilson LR (2013). Attitudes and culture: anunderstanidng of the factors that influence the functioning of accountability mechanisms in primary health care settings. BMC Health Serv Res.

[7] Choudhary GB, Puranik S (2014). A study on employee performance appraisal in health care. Asian J Manag Sci.

[8] Omeheng FLK (2009). Constraints in the implementation of performance Management Systems in Developing Countries: the Ghanaian case. Int J Cross-cult Manag.

[9] Coovadia H, Jewkes R, Barron P, Sanders D, McIntyre D (2009). The health and health system of South Africa: historical roots of current public health challenges. Lancet.

[10] O'Malley P. Truth and Reconciliation: The Homelands from 1960 to 1990. Chapter 5. Truth and Reconciliation Commission of South Africa Report. 1998;2.

[11] Jobson M. Structure of the health system in South Africa. Khulumani Support Group. 2015. https://www.khulumani.net/active.../225_30267364dfc1416597dcad919c37ac71.html. Accessed 18 June 2018.

[12] Kautzky K, Tollman SM (2008). A Perspective on Primary Health Care in South Africa. South African Health Review. Chapter 2.

[13] Pillay Y, McCoy D, Asia B (2001). The district health system in South Africa: progress made and next steps.

[14] O'Neill JW, Beauvais LL, Scholl RW (2001). The use of organizational culture and structure to guide strategic behavior: an information processing perspective. J. Behav. Appl. Manag.

[15] Dorros GL (2006). Building management capacity to rapidly scale up health services and outcomes.

[16] Kotter J. What leaders really do. Harv Bus Rev. 2001; 10104518

[17] World Health Organisation (2005). Strengthening Management in low-Income Countries.

[18] Kwamie A (2015). Balancing Management and Leadership in complex health systems: comment on management matters: a leverage point for health systems strengthening in Global Health. Int J Health Policy Manag.

[19] von Holdt Karl (2010). Nationalism, Bureaucracy and the Developmental State: The South African Case. South African Review of Sociology.

[20] Maphunye K (2002). The features of South Africa’s post-1994 civil service and the challenges it faces in civil service and the challenges it faces in the new dispensation. African Administrative Studies.

[21] Schien E (1995). Organisational Culture and Leadership.

[22] Konteh FH, Mannion R, Davies H (2008). Clinical governance views on culture and quality improvement. Clinical Governance: An International Journal.

[23] Parmelli E, Flodgren G, Beyer F, Baillie N, Schaafsma ME, Eccles MP (2011). The effectiveness of strategies to change organisational culture to improve healthcare performance: a systematic review. Implement Sci.

[24] Pillay R (2008). The skills gap in Hospital Management in the South African Public Health Sector. J. Public Health Manag. Pract.

[25] National Planning Commission. National Development Plan 2030. Our Future - make it work. National Planning Commission. Pretoria - Republic of South Africa: The Presidency; 2012.

[26] Public Service Commission (2011). Report on the implementation of the Performance Management and Development System for Senior Managers in the Western Cape Province.

[27] Gilson L, Elloker S, Olckers P, Lehmann U (2014). Advancing the application of systems thinking in health: south African examples of a leadership of sensemaking for primary health care. Health Research Policy and Systems.

[28] Martinez J, Martineau T. Introducing performance Management in National Health Systems: issues on policy and implementation. An IHSD Issues Note. 2001;

[29] Vasan A, Mabey DC, Chaudhri S, Epstein HB, Lawn SD (2017). Support and performance improvement for primary health care workers in low- and middleincome countries: a scoping review of intervention design and methods. Health Research Policy and Planning.

[30] Silva P, Ferreira A (2010). Performance management in primary healthcare services: evidence from a field study. Qual Res Account Manag.

[31] Eldenburg L, Hermalin BE, Weisbach MS, Wosinska M (2004). Governance, performance objectives and organizational form: evidence from hospitals. J Corp Finan.

[32] McClure ML, Poulin MA, Sovie MD, Wandelt MA (1983). Magnet Hospitals: Attraction and retention of professional nurses.

[33] Nyikuri M, Tsofa B, Barasa E, Okoth P, Molyneux S (2015). Crises and resilience at the frontline: public health facility managers under devolution in a Sub-County on the Kenyan coast. PLoS One.

[34] Gilson L, Barasa E, Nxumalo N, Cleary S, Goudge J, Molyneux S, Tsofa B, Lehmann U (2017). Everyday resilience in district health systems: emerging insights from the front lines in Kenya and South Africa. BMJ Global Health.

[35] Statistics South Africa. Census 2011. http://www.statssa.gov.za/?page_id=3839. 2011. Accessed 15 May 2017.

[36] Kalombo G (2005). Understanding political corruption in post-apartheid South Africa: the Gauteng experience (1994–2004). Faculty of Humanities.

[37] Murray MJ (2011). City of extremes: the spatial politics of Johannesburg. Durham and.

[38] Department of Public Service and Administration (2016). Annual report on employment equity in the public service 2015/16.

[39] Chopra M, Daviaud E, Pattinson R, Fonn S, Lawn JE (2009). Saving the lives of South Africa’s mothers, babies, and children: can the health system deliver?. Lancet.

[40] Munyewende PO, Rispel LC (2014). Using diaries to explore the work experiences of primary health care nursing managers in two south African provinces. Glob Health Action.

[41] Egger D, Travis P, Dolvo D, Hawken L (2007). Strengthening management in low-income countries.

[42] Yin RK (2003). Case study research: design and methods.

[43] Green A (2007). An introduction to planning for developing health systems.

[44] Luthuli TB (2009). From compliance to performance: a leadership challenge in the South African public service. J Public Adm.

[45] Department of Public Service and Administration (2007). Employee performance management and development system.

[46] Codjoe HM, Tettey W, Puplampu KP, Berman BJ (2003). Is Culture the obstacle to development in Ghana? A critique of the culture-development thesis as it applies to Ghana and South Korea. Critical perspectives on politics and socio-economics in Ghana.

[47] Ehtesham UM, Muhammad TM, Muhammad SA (2011). Relationship between organizational culture and performance management practices: a case of University in Pakistan. J Competitiveness.

[48] von Holdt K, Maserumule B, Webster E, von Holdt K (2005). After Apartheid: Decay or Reconstruction in a Public Hospital?. Beyond the Apartheid Workplace: Studies in Transition.

[49] Mendonca M, Kanungo RN (1996). Impact of culture on performance Management in Developing Countries. Int J Manpow.

[50] Hofstede G (1981). Motivation, leadership, and organization: do American theories apply abroad?. Organ Dyn.

[51] Leon N, Bhunu F, Kenyon C (2001). Voices of Facility Managers. Chapter 11. South African Health Review.

[52] Morgan P. The idea and practice of systems thinking and their relevance for capacity development: European Centre for Development Policy Management; 2005.

[53] Buchan J (2004). What difference does ("good") HRM make?. Hum Resour Health.

